# Relationships of Symptom Groups and Functioning Domains in Patients with Advanced-Stage Non-Small Cell Lung Cancer Undergoing Treatment: A Cross-Sectional Study

**DOI:** 10.3390/healthcare9010028

**Published:** 2020-12-30

**Authors:** Myung Kyung Lee

**Affiliations:** College of Nursing, Research Institute of Nursing Science, Kyungpook National University, Daegu 41944, Korea; mlee@knu.ac.kr

**Keywords:** fatigue, functioning, lack of energy, lung cancer, pain, quality of life, symptom

## Abstract

The purpose of this study was to describe the symptoms experienced by patients with non-small cell lung cancer (NSCLC), examine whether different symptom groups significantly affected different functioning domains in these patients, and determine the effect of the “lack of energy” and “pain” symptom groups on the different functioning domains of health-related quality of life (HRQOL). From a single tertiary institution, this cross-sectional study enrolled 135 consecutive NSCLC patients who were mostly undergoing chemotherapy and were in the advanced stage (National University Hospital, Daegu, South Korea). Clinical and self-reported demographic information and data on different functioning domains (from the European Organisation for Research and Treatment of Cancer Quality of Life Questionnaire C30 (EORTC QLQ-C30)), symptom experience (from the EORTC QLQ-LC13), and the Symptom Distress Scale (SDS) were examined. The four most common symptoms were fatigue (69%), pain (47%), dyspnea (38%), and lack of appetite (36%). The “pain” symptom group was negatively associated with physical, emotional, cognitive, and role functioning. The “lack of energy” symptom group was negatively associated with physical, role, emotional, social, and cognitive functioning. The “lack of energy” symptom group explained the most variance for physical and role functioning, and the “pain” symptom group explained the most variance for emotional functioning. Impaired concentration explained the most variance for cognitive functioning.

## 1. Introduction

Lung cancer (LC) is the most common cause of cancer-related deaths, and accounts for approximately 20% of all cancer-related deaths worldwide. Despite improvements in diagnosis and treatment, only 10.9% of individuals with LC live five years or more [1]. The five-year relative survival rates for LC only increased by 1% (18% to 19%) from 2004 to 2014 in the United States [2,3] and by 8.5% (19.7% to 28.2%) from 2006 to 2012 in South Korea [4,5]. LC is also associated with a higher symptom burden than other cancers [6]. The unique symptom profile and poor prognosis can lead to great distress in patients with LC [7] and to poor health-related quality of life (HRQOL) [6,8], which is an established prognostic indicator of overall survival [9]. For these reasons, identification of symptoms that significantly influence HRQOL is crucial for LC management.

Fatigue may co-occur with dyspnea in patients with LC, and this co-occurrence is associated with “lack of energy” [10]. Additionally, many patients receiving treatment for LC experience lack of appetite, diarrhea, and dysphagia [11], and these symptoms can cause undernourishment [12], which can further reduce the appetite of these patients [13]. Previous studies identified lack of appetite and fatigue as co-occurring symptoms (a symptom cluster) in patients with LC [14]. In addition, symptoms related to undernourishment are associated with a lack of energy in these patients [15]. A previous qualitative study reported that dyspnea played a primary role in the onset of fatigue in patients with LC [16]. Thus, there is abundant evidence that fatigue, dyspnea, and symptoms related to undernourishment (appetite loss, diarrhea, and dysphagia) are clinically relevant in patients with LC. Further research is needed to investigate the combined effects of fatigue, dyspnea, appetite loss, bowel disruptions, and dysphagia, which together lead to “lack of energy”, when considering their impact on HRQOL.

The pain in patients with LC can be caused by the tumor itself, treatment, diagnostic tests, surgeries or procedures (e.g., biopsy, puncture, mediastinoscopy, thoracotomy), local or metastatic invasion of a tumor into the chest and consequent inflammation or pulmonary embolism of the invaded area, as well as pressure on bones, nerves, or other organs [17]. More specifically, invasion of a tumor into the chest causes chest pain, which is characterized by increased respiratory movements and neuropathic pain [18]. Taxane- or platinum-based chemotherapy can cause peripheral neuropathy or a burning sensation at the injection site. Radiotherapy can cause skin irritation and pain. Sore mouth following chemotherapy or radiation therapy is also common. Thus, the pain experienced by patients with LC includes site-specific pain (in the chest, arm and shoulder, or other parts) and peripheral neuropathy. Research should investigate the combined effects of pain in specific regions, peripheral neuropathy, and sore mouth when considering the impact of pain on HRQOL.

Previous studies of LC have examined the relationship of the single symptom of fatigue and/or pain with HRQOL [19] and the relationships of clusters of symptoms with HRQOL [10,20]. There is abundant evidence that patients with LC experience a symptom cluster [15,20]. For example, pain and fatigue commonly co-occur as a symptom cluster [21]. However, different studies have reported different symptom clusters in patients with LC, probably due to discrepancies in methodologies (statistical methods, tools for cluster identification, symptom assessment tools) and patient characteristics [22]. Hence, there is no generally accepted characterization of symptom clusters in LC [23]. This led us to examine whether symptom groups, rather than symptom clusters, influence HRQOL in patients with LC. The symptom group in this study refers to a group of clinically relevant symptoms based on previous studies, whereas the symptom cluster is two or more co-occurring symptoms [24].

Thus, we first classified the symptoms that significantly impacted HRQOL into two groups—a “lack of energy” group and a “pain” group—and then examined how these symptom groups affected patient HRQOL during treatment for non-small cell lung cancer (NSCLC). The results of this study may help clinicians in improving the HRQOL of patients undergoing NSCLC treatment and providing a better understanding of the significance of the different symptom groups on HRQOL in these patients.

The specific purposes of this study were to (a) describe the symptoms experienced by patients with NSCLC; (b) determine whether different symptom groups significantly affected the functioning domains of patients with NSCLC; and (c) determine the contribution of each symptom group (“lack of energy” and “pain”) to the different functioning domains of HRQOL.

## 2. Materials and Methods

### 2.1. Study Design and Participants’ Recruitment

This cross-sectional study enrolled 135 consecutive patients who were undergoing treatment for NSCLC at a single tertiary National University Hospital (652 beds) that specializes in cancer and elder health-care in Daegu, South Korea. Quota sampling was used to match the most recently reported male/female incidence ratio of LC in South Korea (new male cases: 17,790 [69%]; new female cases: 7990 [31%]) [5]. Patients were eligible if they were at least 20 years old, diagnosed with NSCLC as the primary cancer, undergoing non-surgical treatments, understood the study, and had sufficient reading and communication skills to answer the questionnaires. Patients were excluded if they were psychologically unstable, had a cognitive or sensorimotor dysfunction, or had a delay of treatment due to side effects, such as pneumonia or unconsciousness. Potentially eligible patients were identified by review of medical information in the hospital registry and interviews.

A review of the hospital electronic medical records indicated that 168 patients were potentially eligible from June 2017 to January 2018. Following clinical appointments, research staff personally contacted these patients at an outpatient clinic or in the admission ward and provided detailed information on the study and the right to refuse participation at any time. All patients had the opportunity to ask any questions about the study. Among 168 potentially eligible patients, 31 (18.5%) refused to participate. The most common reasons for refusal were concerns about privacy violation and the patient’s family did not want them to participate in the study. After a patient agreed to participate and signed the written informed consent agreement, a study questionnaire was distributed at the outpatient and inpatient facilities. All remaining 137 patients (81.5%) agreed to participate in the study, but 2 patients had incomplete responses on the main variables, and were excluded. Data from 135 patients (80.4%) were included in the statistical analyses.

### 2.2. Data Collection

After signing the informed consent agreements, participants were asked to complete the questionnaire (a paper-based self-report). Outpatients completed the questionnaire immediately after recruitment, and inpatients were given more time to complete the questionnaire. Participants read and answered the questionnaire by themselves. Clinical data (comorbidities, NSCLC stage, time since diagnosis, current treatment, prior treatment, histological type) were collected by review of the hospital’s electronic medical records. A research staff contacted patients by telephone if an incomplete response or missing data were noted in the questionnaire. The present study was the second study of a large project consisting of two small studies. The first study examined a multiple mediation model of spiritual well-being and will be published elsewhere [25]. The participants were recruited for both studies. Moreover, both studies were performed following the tenets of the Declaration of Helsinki and the study protocol was approved by the institutional review boards of National University and Hospital (KNUMC 2017-04-004, KNU 2017-45), Daegu, South Korea.

### 2.3. Measurements

The following instruments were used: (a) a self-reported demographic questionnaire, (b) the functional scale of the European Organisation for Research and Treatment of Cancer (EORTC) Quality of Life Questionnaire C30 (QLQ-C30), (c) the LC-specific symptom scale of the EORTC QLQ-LC13 module, and (d) the Symptom Distress Scale (SDS). The demographic questionnaire collected information on sex, age, marital status, caregiver characteristics, job status, monthly income, religion, educational level, and type of national health insurance. The clinical information included comorbidities, NSCLC stage and histological type, time since diagnosis, and current and prior treatments.

#### 2.3.1. Functioning

Functioning in different domains was measured using the EORTC QLQ-C30 [26]. This instrument is a brief, internationally validated, self-reported, 30-item questionnaire that assesses cancer-specific quality of life (QOL). This questionnaire has 5 subscales that measure functioning (physical, social, role, cognitive, and emotional), 9 symptom subscales (fatigue, nausea/vomiting, pain, dyspnea, sleep disturbances, appetite loss, constipation, diarrhea, and financial impact), and a global QOL scale. The Korean versions of the EORTC QLQ-C30 were previously validated [27]. Cronbach’s α coefficient in this study sample was 0.88 for all subscales in the EORTC QLQ-C30.

#### 2.3.2. Symptoms

All symptoms specific to LC (cough, hemoptysis, dyspnea, and pain in the chest, arm and shoulder, and other parts), to cancer in general (nausea, loss of appetite, insomnia, fatigue, bowel disruptions, impaired concentration), and related to treatment (sore mouth, dysphagia, peripheral neuropathy, and alopecia) were recorded.

LC-specific symptoms and treatment-related side effects were assessed using the EORTC QLQ-LC13 module. This module has 13 questions, each rated using a 4-point Likert scale which ranges from 1 (not at all) to 4 (very much), and is designed for use by patients receiving chemotherapy and/or radiotherapy. The LC-associated symptoms are cough, hemoptysis, and dyspnea; site-specific pain includes pain in the chest, arm and shoulder, and other parts. Treatment-related side effects are sore mouth, dysphagia, peripheral neuropathy, and alopecia. In this study, pain refers to pain in the chest, arm and shoulder, or other parts. The EORTC QLQ-LC13 module was validated at two international multicenter studies and has adequate reliability and validity [28]. Cronbach’s α coefficient in this study sample was 0.81 for all subscales in the EORTC QLQ-LC13.

General cancer symptoms were measured using the SDS [29,30]. The SDS is a widely used self-rating cancer symptom scale with 13 items, each rated using a 5-point Likert scale which ranges from 1 (no symptoms) to 5 (very severe symptoms). This scale determines patient symptom experiences based on how they were feeling recently. The following 7 of 13 items in this scale were used: nausea (frequency), nausea (intensity), loss of appetite, insomnia, fatigue, bowel disruptions, and lack of concentration; the other 6 items overlapped with items in the EORTC QLQ-LC13. The average score of nausea frequency and nausea intensity was scored as a single “nausea” score. Cronbach’s α coefficient in this study sample was 0.73 for all scales in the SDS.

All scale and item scores for the EORTC QLQ-C30, QLQ-LC13, and SDS were linearly converted to ranges of 0 to 100 using the EORTC-QLQ-C30 scoring manual [31]. A high score for the functional subscale in the EORTC QLQ-C30 indicated higher functioning. High scores for the symptom subscale of the EORTC QLQ-LC13 and of the SDS indicated greater symptom burden.

### 2.4. Statistical Analyses

Sociodemographic data, clinical characteristics, and symptoms are presented as numbers and frequencies using descriptive statistics. The occurrences of symptoms were estimated as follows. The symptom score >33 of EORTC QLQ-LC13 and ≥25 of SDS were considered to have the symptom. To find out confounders on the relationship between symptoms and functioning, the relationships of sociodemographic and clinical characteristics with the presence of 14 symptoms were determined using the chi-squared test. The associations between treatment modality and functioning domains were tested using one-way analysis of variance and Scheffe’s test. The relationships of functioning with the presence of symptoms were analyzed using analysis of covariance (ANCOVA) to control the confounders, and the general linear model was adjusted for age, marital status, caregiver status, practicing a religion, job status, monthly household income, education level, type of health insurance, comorbidities, time since diagnosis, NSCLC stage, and current and prior treatments. The results of the ANCOVA are presented as least-squares means (LSmeans). The associations of functioning with two symptom groups (“pain” and “lack of energy”) were determined. The pain symptom group was the sum of 5 symptoms in different regions: chest; arm and shoulder; other parts; peripheral neuropathy; and mouth. The lack of energy symptom group was the sum of 5 symptoms: dyspnea, lack of appetite, bowel pattern, dysphagia, and fatigue.

Stepwise multiple regression analyses were used to identify the symptom group that had the greatest impact on each functioning scale of the HRQOL. The multiple regression model included the variables of “pain” and “lack of energy” symptom groups and all sociodemographic and clinical characteristics as independent variables. The results of the stepwise multiple regression analyses were partial R^2^, β coefficient, *p*-value, and model R^2^. Statistical analyses were two sided; *p*-values less than 0.05 were considered statistically significant. All statistical analyses were conducted using SAS version 9.4 (SAS Institute, Cary, NC, USA).

## 3. Results

### 3.1. Characteristics of Participants

Approximately 70% of the patients were male, the overall mean age was 66 years, and 93% of the patients were married (Table 1). Approximately 60% of the patients completed middle school or had lower education levels. Most patients had advanced-stage LC (stage III, 28%; stage IV, 60%) and were undergoing treatment, and 75% of the patients were undergoing chemotherapy. The average time since NSCLC diagnosis was 15 months (range, 1–90 months) (Table 1).

### 3.2. Symptoms and Symptom Groups

**Figure 1:**
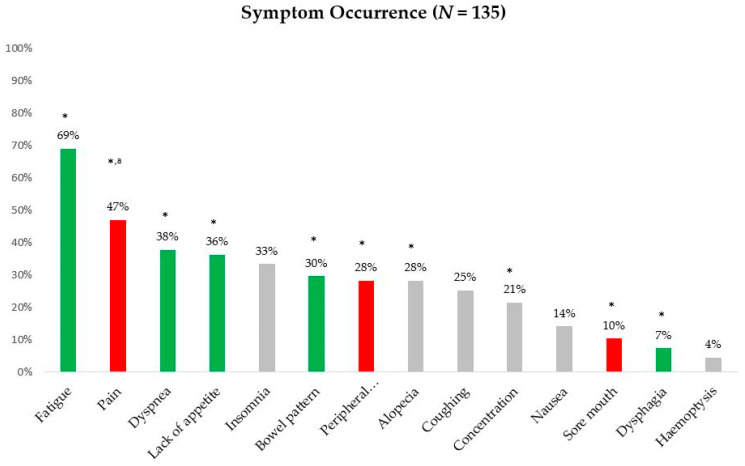
Occurrence of lung cancer (LC) symptom. Green bar, lack of energy symptom group; red bar, pain symptom group. * Statistically significant at *p* < 0.05 in the association between each symptom and functioning domains. ^a^ Pain indicated reports of pain in the chest, arm and shoulder, and other parts.

The four most common symptoms were fatigue (69%), pain (47%), dyspnea (38%), and lack of appetite (36%) (Figure 1).

### 3.3. Association of Sociodemographic and Clinical Characteristics with Symptom Experience

Women experienced more severe fatigue than men (*p* = 0.035). Patients without a spouse experienced more severe lack of appetite (*p* = 0.021); impaired concentration (*p* = 0.037); pain in the chest (*p* = 0.022), arm and shoulder (*p* = 0.036), and other parts (*p* = 0.049); dysphagia (*p* = 0.027); hemoptysis (*p* = 0.005); and dyspnea (*p* = 0.041). Patients with less education experienced more severe pain in the arm and shoulder (*p* = 0.028) and dysphagia (*p* = 0.048). Patients enrolled in Korea Medical Aid experienced more severe pain in the chest (*p* = 0.0003) and dysphagia (*p* = 0.041). NSCLC stage was associated with lack of appetite (*p* = 0.016) and insomnia (*p* = 0.032). Patients currently undergoing different types of treatments had differences in lack of appetite (*p* = 0.041), impaired concentration (*p* = 0.005), nausea (*p* = 0.006), bowel pattern (*p* = 0.047), and pain in the chest (*p* = 0.048). Patients who previously received different types of treatments had differences in concentration (*p* = 0.005), nausea (*p* = 0.0003), and peripheral neuropathy (*p* = 0.047). Histological type of LC was associated with differences in lack of appetite (*p* = 0.023), coughing (*p* = 0.001), dyspnea (*p* = 0.001), and peripheral neuropathy (*p* = 0.006) (Table 1).

### 3.4. Association of Treatment Modality with Functioning

Role and cognitive functionings were significantly different according to the currently undergoing (*p* = 0.038) and the prior (*p* = 0.020) treatment types, respectively (Table 2).

### 3.5. Differences in Functioning According to Symptoms and Grouping of Symptoms

The symptoms that significantly affected at least one functioning scale were pain in the chest, arm and shoulder, and other parts, peripheral neuropathy, sore mouth, dyspnea, lack of appetite, bowel disruption, fatigue, dysphagia, impaired concentration, and alopecia (Table 3, Figure 1). We classified all symptoms that were relevant to functioning into two groups (“pain” and “lack of energy”) and two single factors (“impaired concentration” and “alopecia”). The “pain” group included pain in the chest, arm and shoulder, and other parts, peripheral neuropathy, and sore mouth. The “lack of energy” group included dyspnea, lack of appetite, bowel disruption, dysphagia, and fatigue.

Analysis of the “pain” group indicated that patients who experienced no pain in the chest, pain in the arm and shoulder, pain in other parts, and no peripheral neuropathy had higher physical functioning (*p* = 0.015, *p* = 0.014, *p* = 0.001, and *p* = 0.009, respectively) and emotional functioning (*p* < 0.0001, *p* < 0.0001, *p* = 0.005, and *p* = 0.001, respectively). Patients who experienced no pain in the arm and shoulder and other parts and no peripheral neuropathy had higher cognitive functioning (*p* = 0.002, *p* < 0.0001, and *p* = 0.012, respectively). Patients who experienced no pain in other parts, no peripheral neuropathy, and no sore mouth had higher role functioning (*p* = 0.008, *p* = 0.004, and *p* = 0.044, respectively). Patients who did not have a sore mouth had better physical functioning (*p* = 0.005).

Analysis of the “lack of energy” group indicated that patients who experienced no dyspnea and no lack of appetite had higher physical functioning (*p* < 0.0001 and *p* = 0.002, respectively), role functioning (*p* < 0.0001 and *p* = 0.027, respectively) and emotional functioning (*p* = 0.005 and *p* = 0.003, respectively). Dyspnea adversely affected social functioning (*p* = 0.026), and lack of appetite adversely affected cognitive functioning (*p* = 0.011). Patients who experienced no bowel disruption had higher physical functioning (*p* = 0.047), role functioning (*p* = 0.049), and cognitive functioning (*p* = 0.036). Dysphagia was associated with reduced cognitive functioning (*p* = 0.027), and fatigue was associated with reduced emotional functioning (*p* = 0.011).

Analysis of the two single factors indicated that impaired concentration was associated with decreased physical functioning (*p* = 0.010), role functioning (*p* = 0.008), emotional functioning (*p* = 0.018), and cognitive functioning (*p* < 0.0001). Alopecia was associated with decreased emotional functioning (*p* = 0.014).

### 3.6. Factors Affecting Functioning in Patients Undergoing Treatment for NSCLC

Higher physical functioning was negatively associated with the “lack of energy” symptom group (R^2^ = 22%, β = −7.5, *p* < 0.001), the “pain” symptom group (R^2^ = 4%, β = −4.9, *p* < 0.01), and receiving immunotherapy (R^2^ = 6%, β = −30, *p* < 0.05). Higher physical functioning was positively associated with more education (R^2^ = 3%, β = 13.6, *p* < 0.01) and having a job (R^2^ = 2%, β = 11.1, *p* < 0.05). Higher role functioning was negatively associated with the “lack of energy” symptom group (R^2^ = 18%, β = −11.6, *p* < 0.0001) and impaired concentration (R^2^ = 3%, β = −16.7, *p* < 0.05). Higher emotional functioning was negatively associated with the “pain” symptom group (R^2^ = 29%, β = −12.2, *p* < 0.0001) and receiving radiotherapy (R^2^ = 2%, β = −16.3, *p* < 0.05). Higher emotional functioning was positively associated with having a job (R^2^ = 3%, β = 14.5, *p* = 0.041). Higher cognitive functioning was negatively associated with the “pain” symptom group (R^2^ = 5%, β = −3.7, *p* < 0.01) and impaired concentration (R^2^ = 28%, β = −25.4, *p* < 0.0001). Higher social functioning was negatively associated with the “lack of energy” symptom group (R^2^ = 5%, β = −7.5, *p* < 0.001), but positively associated with greater age (R^2^ = 9%, β = 1.2, *p* < 0.01) and higher income (R^2^ = 7%, β = 14.1, *p* < 0.05).

The model R^2^ values were 37% for physical functioning, 21% for role functioning, 34% for emotional functioning, 33% for cognitive functioning, and 21% for social functioning. The “lack of energy” symptom group explained the most variance for physical functioning (22%) and role functioning (18%), and the pain symptom group explained the most variance for emotional functioning (29%). Impaired concentration explained the most variance for cognitive functioning (28%) (Table 4).

## 4. Discussion

The purpose of the current study was to determine the relationships of symptom groups with functioning domains of patients who had NSCLC and were undergoing treatment. For patients undergoing treatment, the four most common symptoms were fatigue, pain, dyspnea, and lack of appetite. Considering the significant relationship between symptoms and HRQOL, we established two symptom groups (“lack of energy” and “pain”) and one single symptom (“impaired concentration”). The “lack of energy” symptoms were associated with physical, role, and social functioning and explained the most variance for physical and role functioning. ”Pain” symptoms were associated with physical, emotional, and cognitive functioning and explained the most variance for emotional functioning. Impaired concentration explained the most variance for cognitive functioning.

Our results indicated that the four most common symptoms experienced by patients with NSCLC during treatment were fatigue, pain, dyspnea, and lack of appetite. This result is supported by previous studies that examined patients with advanced LC who were undergoing treatment or palliative care [32]; patients with LC who reported fatigue, pain, and dyspnea were the most common symptoms [33,34]; patients with LC during chemotherapy who reported the most common symptoms were lack of energy, lack of appetite, and pain [6]; women with advanced stage of NSCLC receiving chemotherapy who reported the most common symptoms were fatigue, dyspnea, anorexia, and pain [20]; and patients with advanced LC, more than 90% of whom reported experiencing pain and fatigue [19].

We found that fatigue was one of the most common symptoms, consistent with previous studies of patients with advanced-stage NSCLC [35]. However, the prevalence of fatigue in our study (69%) differed slightly from that of previous studies. Previous studies reported prevalence rates of approximately 57% for LC survivors after thoracotomy or lobectomy [36], approximately 60% for patients with early-stage LC [37], and 65% for women with advanced-stage NSCLC who were receiving chemotherapy [20]. The difference may be that the patients in this study were lumped together at different stages of the NSCLC. However, the other studies had specific populations. These small differences may be also attributed to the differences in the receipt of current or prior treatment, measurement tools, and patient characteristics. Our NSCLC patients mostly had advanced-stage cancer and were undergoing treatment, and the prevalence of fatigue in our subjects was similar to that reported for cancer patients overall, patients with LC after invasive surgery and chemotherapy, patients with early-stage or advanced-stage cancer, and patients in palliative care.

After fatigue, we found that pain (47%) and dyspnea (38%) were the symptoms with the highest prevalence rates. This result is similar to that of previous studies, which reported a prevalence rate of 49% for pain [20] and 39% for dyspnea [34].

Overall, our results showed that the “lack of energy” and “pain” symptom groups and impaired concentration negatively influenced HRQOL, in line with previous studies that reported negative relationships between symptom distress and HRQOL [6,8]. Among the five HRQOL functioning domains, the “lack of energy” symptom group had the greatest influence on physical functioning (explaining 59% of the variance), role functioning (explaining 86% of the variance), and social functioning (24% of the variance). This result is in line with a previous study of patients with advanced-stage NSCLC, which reported that the fatigue/anorexia cluster had the greatest impact on physical and role functioning [15]. Most of our patients (88%) had advanced-stage NSCLC and were undergoing treatment. Thus, reduced functioning caused by a lack of energy appears to be common in patients with NSCLC who are undergoing treatment, patients with advanced-stage NSCLC. Our findings indicated that the impact of “lack of energy” on role, physical, and social functioning overwhelmed the impact of the other functional subscales.

Recent studies of LC have not sufficiently described the relationship between lack of energy and social functioning. However, previous research reported that severe fatigue seriously impaired engagement at work and participation in social and family activities, activities of daily living, self-care, and hobby and leisure activities [33,38,39]. Therefore, “lack of energy” negatively influences physical, role, and social functioning.

We classified lack of appetite, bowel disruption, and dysphagia in the “lack of energy” symptom group. Our finding that the “lack of energy” symptom group influenced physical, role, and social functioning is consistent with a previous study that reported that undernourished patients had worse physical, social, and role functioning [12].

The “pain” symptom group had the greatest influence on emotional functioning, but had less influence on physical and cognitive functioning. This result is consistent with several previous studies of patients with LC, which reported that the pain cluster [15] and pain [19] had strong influences on the emotional and physical functioning scales of HRQOL. The physiological response to pain can lead to anxiety and sadness, and depression or anxiety can also lead to increased pain [40]. A previous study of patients with LC reported that having more emotional problems led to more frequent pain and greater pain severity [41]. Other research found that the perceived level of pain was lower in patients who received psychological interventions [42]. Thus, among the several HRQOL functional domains, experience of pain has a significant negative impact on emotional functioning.

Most of our patients were receiving chemotherapy and had advanced-stage NSCLC, and 28% of them reported peripheral neuropathy. Peripheral neuropathy was in the “pain” symptom group, and affected physical and emotional functioning. Peripheral neuropathy is common in patients taking taxane- and platinum-based agents, which are commonly used by patients with advanced-stage NSCLC [43]. Generally, peripheral neuropathy is associated with a decreased HRQOL in patients with NSCLC [44] and has a particularly strong impact on ambulation [15]. Peripheral neuropathy may decrease the ease of ambulation and interfere with daily activities, and this could cause a deterioration of physical and emotional functioning.

Few previous studies of patients with LC reported a relationship between pain and cognitive functioning. However, previous studies of patients with cancer found that patients who had more pain had impaired cognitive functioning [41,45,46,47]. According to the previous studies, sustained pain, use of analgesics, and reporting more incidents or greater severity of pain are all associated with decreased cognitive functioning.

Impaired concentration was a single symptom that had the strongest influence on cognitive functioning. Cognitive functioning included the following domains: attention and concentration, language (e.g., difficulties with finding the right word), speed of processing (e.g., multitasking), memory (e.g., remembering new information), and the effort and time needed to accomplish these tasks [48]. These results thus indicate that impaired attention and concentration correlate with decreasing cognitive functioning in cancer survivors.

As in our study, some previous studies showed associations between more education and higher physical functioning among cancer survivors [49,50]. Educational level is an indicator of social position, occupation, and income [49], and reflects better access to resources that may improve a patient’s ability to cope with cancer [51].

Previous research reported associations of employment with higher physical [52] and social [53] functioning. Cancer patients with occupations have a source of economic support during recovery, and this may lead to their better HRQOL. A cancer patient with a job may have a reduced financial burden from treatment to recovery and may also be able to afford additional therapy. Lost productivity at work is associated with worse social functioning in patients with thyroid cancer [53]. Higher income was also associated with improved social functioning [54]. Having more education and a higher income are not modifiable factors following a diagnosis of cancer; however, coping strategies tailored to these groups may help to ease the impact of LC treatment and improve the quality of survivorship.

There is a widespread need to improve the functioning and alleviate the symptoms of patients with NSCLC. Symptom monitoring allows earlier detection of symptoms, adverse events, and their recurrence, so that interventions can be used to improve HRQOL and possibly prolong survival [55].

The “lack of energy” group and the “pain” group affected different HRQOL functioning domains. It is important for oncology nurses to consider symptom groups, because the symptoms in a specific group had highly correlations with each other. In particular, patients can simply or generally express if they lack energy or feel tired or ill, but they have more difficulty in describing the experience of individual symptoms, such as dysphagia, diarrhea, anorexia, and peripheral neuropathy. Thus, an oncology nurse needs to understand a patient’s general symptoms and specific symptoms. When an oncology nurse critically analyzes the symptoms of a patient with LC, then comprehensive and preventive interventions that may improve the different HRQOL functioning domains can be applied. Nursing care based on symptom groups, monitoring of specific symptoms, and assessing disruptions of different functioning domains in daily life may facilitate the application of interventions that improve functioning and extend survival. We suggest that oncology nurses should give special attention to the HRQOL functioning domains of their patients by considering the two symptom groups identified here, namely “lack of energy” and “pain”.

This study has several limitations because we used consecutive sampling of patients with NSCLC who were undergoing therapy in a single hospital that specializes in cancer. We made an effort to reduce selection bias by the use of consecutive sampling and matching of the LC incidence ratio of men and women in South Korea to reduce confounding bias due to sex. Moreover, because of the cross-sectional design of this study, our results only suggest the existence and factors that affect functioning at one point in time in a group of patients with NSCLC who were undergoing standard therapy. We cannot infer causal relationships of symptom groups with different functioning domains. Future longitudinal studies are needed to determine whether interventions that change the symptoms identified here alter different functioning domains. Despite these limitations, our study is an important contribution because the results showed that the symptoms of patients with advanced-stage NSCLC influenced different HRQOL functioning domains during the treatment period.

## 5. Conclusions

This research studied patients with mostly advanced stage and receiving chemotherapy and found that the “lack of energy” symptom group was associated with the physical, role, and social functioning and explained the most variance for physical and role functioning. The “pain” symptom group was associated with emotional, physical, and cognitive functioning and explained the most variance for emotional functioning. Impaired concentration explained the most variance for cognitive functioning. These results indicated that different symptom groups had different effects on the functioning of patients with NSCLC who were undergoing treatment.

## Figures and Tables

**Table 1:** Association between sociodemographic and clinical characteristics and symptom experience.

		Symptom Distress Scale, Occurrence, *n* (%)						EORTC QLQ-LC13, Occurrence, *n* (%)									
Variable	*n* (%) *N* = 135	Lack of Appetite	Concentration	Fatigue	Nausea	Insomnia	Bowel Pattern	LCPC	LCPA	LCPO	LCDS	LCHA	LCSM	LCCO	LCDY	LCPN	LCHR
Sex																	
Male	96 (71.1)	34 (35.4)	20 (20.8)	61 (63.5)	13 (13.5)	29 (30.2)	27 (28.1)	27 (28.1)	8 (8.3)	15 (15.6)	7 (7.3)	5 (5.2)	9 (9.4)	26 (27.1)	36 (37.5)	26 (27.1)	27 (28.1)
Female	39 (28.9)	15 (38.5)	9 (23.1)	32 (82.1)	6 (15.4)	16 (41.0)	13 (33.3)	9 (23.1)	3 (7.7)	6 (15.4)	3 (7.7)	1 (2.6)	5 (12.8)	8 (20.5)	15 (38.5)	12 (30.8)	11 (28.2)
*p*	―	0.844	0.819	0.035	0.780	0.227	0.548	0.548	0.902	0.972	0.936	0.672	0.545	0.425	0.917	0.666	0.993
Age, years																	
<65	59 (43.7)	19 (32.2)	17 (28.8)	39 (66.1)	7 (11.9)	22 (37.3)	15 (25.4)	16 (27.1)	4 (6.8)	9 (15.3)	6 (10.2)	4 (6.8)	7 (11.9)	17 (28.8)	21 (35.6)	17 (28.8)	18 (30.5)
≥65	76 (56.3)	30 (39.5)	12 (15.8)	54 (71.1)	12 (15.8)	23 (30.3)	25 (32.9)	20 (26.3)	7 (9.2)	12 (15.8)	4 (5.3)	2 (2.6)	7 (9.2)	17 (22.4)	30 (39.5)	21 (27.6)	20 (26.3)
*p*	―	0.384	0.068	0.538	0.515	0.390	0.346	0.917	0.609	0.932	0.280	0.404	0.616	0.392	0.645	0.880	0.591
Marital status																	
No spouse	10 (7.4)	7 (70.0)	5 (50.0)	8 (80.0)	2 (20.0)	6 (60.0)	3 (30.0)	6 (60.0)	3 (30.0)	4 (40.0)	3 (30.0)	3 (30.0)	2 (20.0)	5 (50.0)	7 (70.0)	3 (30.0)	4 (40.0)
With spouse	125 (92.6)	42 (33.6)	24 (19.2)	85 (68.0)	17 (13.6)	39 (31.2)	73 (29.6)	30 (24.0)	8 (6.4)	17 (13.6)	7 (5.6)	3 (2.4)	12 (9.6)	29 (23.2)	44 (35.2)	35 (28.0)	34 (27.2)
*p*	―	0.021	0.037	0.724	0.576	0.083	0.979	0.022	0.036	0.049	0.027	0.005	0.277	0.121	0.041	1	0.467
Caregiver																	
No	21 (15.6)	7 (33.3)	2 (9.5)	12 (57.1)	2 (9.5)	7 (33.3)	4 (19.1)	6 (28.6)	3 (14.3)	4 (19.1)	2 (9.5)	1 (4.8)	1 (4.8)	7 (33.3)	9 (42.9)	7 (33.3)	3 (14.3)
Yes	114 (84.4)	42 (36.8)	27 (23.7)	81 (71.1)	17 (14.9)	38 (33.3)	36 (31.6)	30 (26.3)	8 (7.0)	17 (14.9)	8 (7.0)	5 (4.4)	13 (11.4)	27 (23.7)	42 (36.8)	31 (27.2)	35 (30.7)
*p*	―	0.759	0.245	0.206	0.514	0.999	0.248	0.830	0.377	0.743	0.687	0.939	0.696	0.413	0.601	0.565	0.186
Having a job																	
No	110 (81.5)	38 (34.6)	24 (21.8)	79 (71.8)	13 (11.8)	37 (33.6)	33 (30.0)	28 (25.5)	10 (9.1)	14 (12.7)	6 (5.5)	3 (2.7)	12 (10.9)	24 (21.8)	42 (38.2)	30 (27.3)	31 (28.2)
Yes	25 (18.5)	11 (44.0)	5 (20.0)	14 (56.0)	6 (24.0)	8 (32.0)	7 (28.0)	8 (32.0)	1 (4.0)	7 (28.0)	4 (16.0)	3 (12.0)	2 (8.0)	10 (40.0)	9 (36.0)	8 (32.0)	7 (28.0)
*p*	―	0.375	0.842	0.123	0.114	0.876	0.843	0.504	0.689	0.057	0.088	0.077	0.999	0.059	0.839	0.635	0.985
Monthly income, US $																	
<2000	110 (81.5)	40 (36.4)	26 (23.6)	78 (70.9)	15 (13.6)	36 (32.7)	33 (30.0)	33 (30.0)	11 (10.0)	18 (16.4)	7 (6.4)	5 (4.6)	11 (10.0)	28 (25.5)	43 (39.1)	30 (27.3)	32 (29.1)
≥2000	25 (18.5)	9 (36.0)	3 (12.0)	15 (60.0)	4 (16.0)	9 (36.0)	7 (28.0)	3 (12.0)	0 (0.0)	3 (12.0)	3 (12.0)	1 (4.0)	3 (12.0)	6 (24.0)	8 (32.0)	8 (32.0)	6 (24.0)
*p*	―	0.973	0.201	0.288	0.754	0.754	0.843	0.066	0.216	0.764	0.393	1	0.767	0.880	0.509	0.635	0.609
Educational level																	
≤Middle school	80 (59.3)	34 (42.5)	17 (21.3)	57 (71.3)	13 (16.3)	28 (35.0)	23 (28.8)	21 (26.3)	10 (12.5)	15 (18.8)	9 (11.3)	4 (5.0)	9 (11.3)	20 (25.0)	32 (40.0)	25 (31.3)	25 (31.3)
≥High school	55 (40.7)	15 (27.3)	12 (21.8)	36 (65.5)	6 (10.9)	17 (30.9)	17 (30.9)	15 (27.3)	1 (1.8)	6 (10.9)	1 (1.8)	2 (3.6)	5 (9.1)	14 (25.5)	19 (34.6)	13 (23.6)	13 (23.6)
*p*	―	0.071	0.937	0.475	0.381	0.620	0.787	0.895	0.028	0.217	0.048	1	0.686	0.952	0.521	0.334	0.334
Type of national health insurance																	
Medical aid	20 (14.8)	8 (40.0)	7 (35.0)	17 (85.0)	2 (10.0)	9 (45.0)	6 (30.0)	12 (60.0)	3 (15.0)	5 (25.0)	4 (20.0)	2 (10.0)	5 (25.0)	4 (20.0)	11 (55.0)	8 (40.0)	7 (35.0)
National health insurance	115 (85.2)	41 (35.7)	22 (19.1)	76 (66.0)	17 (14.8)	36 (31.3)	34 (29.6)	24 (20.9)	8 (7.0)	16 (13.9)	6 (5.2)	4 (3.5)	9 (7.8)	30 (26.1)	40 (34.8)	30 (26.1)	31 (27.0)
*p*	―	0.709	0.111	0.092	0.738	0.231	0.969	0.0003	0.225	0.199	0.041	0.217	0.036	0.563	0.085	0.202	0.460
Comorbidity																	
No	94 (69.6)	34 (36.2)	21 (22.3)	61 (64.9)	13 (13.8)	30 (31.9)	28 (29.8)	22 (23.4)	10 (10.6)	16 (17.0)	9 (9.6)	5 (5.3)	9 (9.6)	27 (28.7)	37 (39.4)	26 (27.7)	28 (29.8)
Yes	41 (30.4)	15 (36.6)	8 (19.5)	32 (78.1)	6 (14.6)	15 (36.6)	12 (29.3)	14 (34.2)	1 (2.4)	5 (12.2)	1 (2.4)	1 (2.4)	5 (12.2)	7 (17.1)	14 (34.2)	12 (29.3)	10 (24.4)
*p*	―	0.963	0.713	0.129	0.902	0.597	0.952	0.194	0.172	0.477	0.146	0.667	0.760	0.152	0.566	0.848	0.521
NSCLC stage																	
I	9 (7.1)	1 (11.1)	1 (11.1)	7 (77.8)	0 (0.0)	4 (44.4)	2 (22.2)	4 (44.4)	1 (11.1)	2 (22.2)	0 (0.0)	0 (0.0)	1 (11.1)	2 (22.2)	4 (44.4)	1 (11.1)	1 (11.1)
II	7 (5.5)	3 (42.9)	4 (57.1)	4 (57.1)	1 (14.3)	2 (28.6)	2 (28.6)	2 (28.6)	0 (0.0)	1 (14.3)	0 (0.0)	0 (0.0)	1 (14.3)	1 (14.3)	3 (42.9)	1 (14.3)	1 (14.3)
III	35 (27.6)	23 (53.5)	9 (20.9)	34 (79.1)	8 (18.6)	21 (48.8)	15 (34.9)	14 (32.6)	3 (7.0)	7 (16.3)	7 (16.3)	4 (9.3)	6 (14.0)	18 (41.9)	20 (46.5)	10 (23.3)	12 (27.9)
IV	76 (59.8)	22 (29.0)	15 (19.7)	48 (63.2)	10 (13.2)	18 (23.7)	21 (27.6)	16 (21.1)	7 (9.2)	11 (14.5)	3 (4.0)	2 (2.6)	6 (7.9)	13 (17.1)	24 (31.6)	26 (34.2)	24 (31.5)
*p*	―	0.016	0.144	0.232	0.521	0.032	0.829	0.308	0.940	0.893	0.095	0.372	0.567	0.022	0.379	0.367	0.578
Time since diagnosis, months																	
<12	73 (54.1)	26 (35.6)	15 (20.6)	50 (68.5)	9 (12.3)	28 (38.4)	21 (28.8)	18 (24.7)	4 (5.5)	10 (13.7)	5 (6.9)	2 (2.7)	7 (9.6)	19 (26.0)	24 (32.9)	16 (21.9)	20 (27.4)
≥12, <24	38 (28.1)	15 (39.5)	10 (26.3)	27 (71.1)	5 (13.2)	10 (26.3)	12 (31.6)	10 (26.3)	3 (7.9)	7 (18.4)	5 (13.2)	4 (10.5)	5 (13.2)	10 (26.3)	19 (50.0)	13 (34.2)	13 (34.2)
≥24	24 (17.8)	8 (33.3)	4 (16.7)	16 (66.7)	5 (20.8)	7 (29.2)	7 (29.2)	8 (33.3)	4 (16.7)	4 (16.7)	0 (0.0)	0 (0.0)	3 (8.3)	5 (20.8)	8 (33.3)	9 (37.5)	5 (20.8)
*p*	―	0.886	0.640	0.931	0.572	0.395	0.952	0.724	0.220	0.798	0.147	0.140	0.745	0.863	0.186	0.209	0.510
Current treatment																	
CCRT	11 (8.2)	8 (72.7)	5 (45.5)	7 (63.6)	6 (54.6)	6 (54.6)	5 (45.5)	5 (45.5)	2 (18.2)	2 (18.2)	2 (18.2)	1 (9.1)	1 (9.1)	4 (36.4)	4 (36.4)	3 (27.3)	3 (27.3)
CT	101 (74.8)	32 (31.7)	15 (14.9)	68 (67.3)	11 (10.9)	29 (28.7)	26 (25.7)	21 (20.8)	6 (5.9)	16 (15.8)	7 (6.9)	5 (5.0)	13 (12.9)	25 (24.8)	34 (33.7)	31 (30.7)	28 (27.7)
RT	18 (13.3)	8 (44.4)	6 (33.3)	13 (72.2)	2 (11.1)	8 (44.4)	5 (27.8)	8 (44.4)	3 (16.7)	3 (16.7)	1 (5.6)	0 (0.0)	0 (0.0)	3 (16.7)	10 (55.6)	2 (11.1)	4 (22.2)
IT	5 (3.7)	1 (20.0)	3 (60.0)	5 (100.0)	0 (0.0)	2 (40.0)	4 (80.0)	2 (40.0)	0 (0.0)	0 (0.0)	0 (0.0)	0 (0.0)	0 (0.0)	2 (40.0)	3 (60.0)	2 (40.0)	3 (60.0)
*p*	―	0.041	0.005	0.541	0.006	0.231	0.047	0.048	0.191	0.801	0.498	0.642	0.401	0.479	0.228	0.305	0.430
Prior treatment																	
No	13 (9.6)	6 (46.2)	0 (0.0)	6 (46.2)	5 (38.5)	5 (38.5)	3 (23.1)	1 (7.7)	0 (0.0)	2 (15.4)	1 (7.7)	1 (7.7)	0 (0.0)	2 (15.4)	4 (30.8)	1 (7.7)	2 (15.4)
CT	91 (67.4)	29 (31.9)	18 (19.8)	64 (70.3)	7 (7.7)	24 (26.4)	28 (30.8)	21 (23.1)	6 (6.6)	12 (13.2)	6 (6.6)	3 (3.3)	12 (13.2)	21 (23.1)	32 (35.2)	30 (33.0)	29 (31.9)
RT	12 (8.9)	5 (41.7)	6 (50.0)	10 (83.3)	1 (8.3)	7 (58.3)	2 (16.7)	6 (50.0)	3 (25.0)	3 (25.0)	1 (8.3)	1 (8.3)	1 (8.3)	5 (41.7)	8 (66.7)	4 (33.3)	4 (33.3)
CCRT	8 (5.9)	6 (75.0)	4 (50.0)	7 (87.5)	5 (62.5)	4 (50.0)	3 (37.5)	3 (37.5)	2 (25.0)	2 (25.0)	2 (25.0)	1 (12.5)	1 (12.5)	3 (37.5)	4 (50.0)	3 (37.5)	3 (37.5)
Surgery	11 (8.1)	3 (27.3)	1 (9.1)	6 (54.6)	1 (9.1)	5 (45.5)	4 (36.4)	5 (45.5)	0 (0.0)	2 (18.2)	0 (0.0)	0 (0.0)	0 (0.0)	3 (27.3)	3 (27.3)	0 (0.0)	0 (0.0)
*p*	―	0.140	0.005	0.168	0.0003	0.105	0.787	0.056	0.052	0.632	0.283	0.304	0.565	0.485	0.229	0.047	0.126
Histological type																	
Adenocarcinoma	79 (67.5)	23 (29.1)	16 (20.3)	54 (68.4)	9 (11.4)	30 (38.0)	22 (27.9)	22 (27.9)	7 (8.9)	8 (10.1)	4 (5.1)	1 (1.3)	8 (10.1)	13 (16.5)	21 (26.6)	17 (21.5)	19 (24.1)
Pleomorphic carcinoma	2 (1.7)	1 (50.0)	1 (50.0)	1 (50.0)	0 (0.0)	0 (0.0)	0 (0.0)	2 (100.0)	0 (0.0)	0 (0.0)	0 (0.0)	0 (0.0)	0 (0.0)	2 (100.0)	2 (100.0)	0 (0.0)	0 (0.0)
Squamous cell carcinoma	36 (30.8)	19 (52.8)	9 (25.0)	24 (66.7)	7 (19.4)	11 (30.6)	12 (33.3)	10 (27.8)	3 (8.3)	10 (27.8)	4 (11.1)	2 (5.6)	4 (11.1)	15 (41.7)	20 (55.6)	18 (50.0)	14 (38.9)
*p*	―	0.023	0.405	0.923	0.448	0.527	0.757	0.153	0.905	0.053	0.354	0.270	0.879	0.001	0.001	0.006	0.188

EORTC QLQ-LC13, the European Organisation for Research and Treatment of Cancer Quality of Life Questionnaire the LC-specific symptom scale; CT, chemotherapy; RT, radiotherapy; CCRT, concurrent chemoradiotherapy; IT, immunotherapy. LCPC, pain in the chest; LCPA, pain in the arm and shoulder; LCPO, pain in other parts; LCDS, dysphagia; LCHA, hemoptysis; LCSM, sore mouth; LCCO, coughing; LCDY, dyspnea; LCPN, peripheral neuropathy; LCHR, alopecia.

**Table 2:** Association between treatment modality and functioning.

Treatment Modality	PF		RF		EF		CF		SF	
	Mean (SD)	*p*	Mean (SD)	*p*	Mean (SD)	*p*	Mean (SD)	*p*	Mean (SD)	*p*
Current treatment										
CCRT	64.8 (27.8)		62.1 (39.5) a		69.7 (35.0)		81.8 (24.1)		69.7 (34.0)	
CT	57.9 (27.7)		72.3 (34.9 b		74.2 (33.1)		85.1 (23.2)		72.3 (32.3)	
RT	50.4 (27.6)		56.5 (43.9) a	0.038 (b > a > c) *	62.0 (38.5)		82.4 (25.2)		64.8 (30.7)	
IT	33.3 (35.9)	0.143	30.0 (44.7) c		85.0 (9.1)	0.431	70.0 (27.4)	0.542	60.0 (54.8)	0.720
Prior treatment										
No	62.1 (28.2)		62.8 (44.7)		78.8 (27.1)		92.3 (16.1)a		68.1 (37.9)	
CT	55.2 (27.8)		71.1 (35.7)		75.2 (32.8)		84.1 (22.5)c		73.8 (31.3)	
RT	42.2 (27.8)		44.4 (42.2)		47.9 (39.6)		65.3 (34.4)b		44.4 (32.8)	
CCRT	66.7 (31.9)		62.5 (37.5)		67.7 (36.6)		81.3 (24.3)c	0.020 (a > c > b) *	77.1 (26.6)	
Surgery	70.7 (25.9)	0.120	78.3 (37.7)	0.170	74.2 (32.0)	0.104	95.0 (15.8)a		68.3 (38.0)	0.063

SD, standard deviation; CT, chemotherapy; RT, radiotherapy; CCRT, concurrent chemoradiotherapy; IT, immunotherapy; PF, physical functioning; RF, role functioning; EF, emotional functioning; CF, cognitive functioning; SF, social functioning. * Grouping from Scheffe’s test.

**Table 3:** Symptoms and their cluster affecting functioning.

Symptom Group	Symptom		LSmean (SE)				
			PF	RF	EF	CF	SF
Pain symptom group	Pain in the chest	No	60.3 (2.7)	70.1 (3.8)	80.1 (3.1)	86.2 (2.4)	69.9 (3.3)
		Yes	46.2 (4.7)	61.5 (6.7)	52.0 (5.5)	77.7 (4.2)	72.6 (5.7)
		*p* *	0.015	0.29	<0.0001	0.096	0.701
	Pain in the arm and shoulder	No	58.4 (2.3)	69.6 (3.3)	76.3 (2.7)	86.0 (2.0)	71.0 (2.8)
		Yes	35.2 (8.8)	46.7 (12.5)	30.3 (10.3)	60.4 (7.7)	66.3 (10.8)
		*p* *	0.014	0.086	<0.0001	0.002	0.679
	Pain in other parts	No	59.8 (2.4)	71.5 (3.4)	76.1 (2.9)	87.4 (2.1)	70.9 (2.9)
		Yes	38.9 (5.8)	47.4 (8.2)	53.6 (7.1)	65.1 (5.0)	69.4 (7.4)
		*p* *	0.001	0.008	0.005	<0.0001	0.856
	Peripheral neuropathy	No	60.5 (2.7)	73.9 (3.7)	78.8 (3.2)	87.4 (2.4)	72.0 (3.3)
		Yes	46.4 (4.4)	52.1 (6.1)	56.7 (5.3)	75.2 (3.9)	67.2 (5.4)
		*p* *	0.009	0.004	0.001	0.012	0.459
	Sore mouth	No	58.9 (2.3)	70.1 (3.3)	73.3 (2.9)	84.5 (2.2)	70.5 (2.88)
		Yes	36.6 (7.3)	47.6 (10.4)	66.4 (9.2)	79.3 (6.7)	71.6 (9.0)
		*p* *	0.005	0.044	0.482	0.471	0.911
Lack of energy symptom group	Dyspnea	No	66.3 (2.6)	77.9 (3.8)	79.0 (3.5)	86.7 (2.6)	75.6 (3.4)
		Yes	40.5 (3.3)	51.0 (5.0)	62.1 (4.5)	79.4 (3.4)	62.6 (4.5)
		*p* *	<0.0001	<0.0001	0.005	0.098	0.026
	Lack of appetite	No	62.2 (2.8)	73.5 (4.0)	79.2 (3.4)	88.1 (2.5)	74.3 (3.4)
		Yes	46.7 (3.8)	57.8 (5.4)	60.9 (4.7)	76.7 (3.4)	64.0 (4.7)
		*p* *	0.002	0.027	0.003	0.011	0.091
	Bowel pattern	No	59.6 (2.7)	72.1 (3.8)	74.1 (3.3)	86.9 (2.4)	73.2 (3.2)
		Yes	49.2 (4.3)	57.6 (6.0)	68.9 (5.3)	77.1 (3.8)	64.5 (5.2)
		*p* *	0.047	0.049	0.419	0.036	0.177
	Dysphagia	No	69.6 (3.3)	72.9 (2.9)	72.9 (2.9)	85.3 (2.1)	71.0 (2.8)
		Yes	45.1 (2.6)	68.5 (11.2)	68.5 (11.2)	66.7 (8.0)	66.5 (10.9)
		*p* *	0.066	0.709	0.709	0.027	0.698
	Fatigue	No	59.1 (4.3)	73.6 (6.0)	83.9 (5.1)	87.4 (3.8)	72.6 (5.1)
		Yes	55.4 (2.8)	65.2 (3.9)	67.5 (3.3)	82.4 (2.5)	69.8 (3.4)
		*p* *	0.493	0.259	0.011	0.293	0.658
Single factor	Concentration	No	59.9 (2.5)	72.7 (3.6)	76.4 (3.1)	90.0 (2.0)	72.9 (3.1)
		Yes	44.1 (5.2)	49.7 (7.3)	58.6 (6.4)	61.8 (4.2)	62.6 (6.4)
		*p* *	0.010	0.008	0.018	<0.0001	0.1675
Single factor	Alopecia	No	58.4 (2.7)	67.4 (3.8)	77.2 (3.3)	86.3 (2.4)	73.5 (3.3)
		Yes	51.8 (4.6)	68.8 (6.4)	60.8 (5.5)	78.0 (4.0)	63.4 (5.4)
		*p* *	0.239	0.85	0.014	0.089	0.124
Not significant	Insomnia	No	56.3 (2.8)	66.4 (3.9)	74.5 (3.4)	84.3 (2.5)	72.4 (3.3)
		Yes	57.0 (4.1)	70.5 (5.7)	68.7 (5.0)	83.3 (3.6)	67.1 (4.9)
		*p* *	0.888	0.563	0.346	0.827	0.392
	Hemoptysis	No	57.1 (2.3)	67.4 (3.2)	72.4 (2.8)	84.9 (2.0)	71.6 (2.7)
		Yes	44.1 (11.7)	76.5 (16.4)	75.9 (14.4)	64.2 (10.3)	46.3 (15.7)
		*p* *	0.281	0.592	0.813	0.053	0.119
	Coughing	No	60.0 (2.6)	69.6 (3.7)	72.4 (3.3)	84.8 (2.4)	72.8 (3.2)
		Yes	46.2 (4.6)	62.3 (6.6)	73.1 (5.8)	81.4 (4.2)	64.4 (5.6)
		*p* *	0.013	0.352	0.928	0.49	0.212
	Nausea	No	57.1 (2.5)	65.7 (3.5)	73.8 (3.0)	85.0 (2.2)	70.9 (2.9)
		Yes	53.1 (6.8)	80.7 (9.5)	65.1 (8.3)	77.5 (6.0)	69.1 (8.3)
		*p* *	0.589	0.15	0.339	0.259	0.841

LSmean, least-squares mean; SE, standard error; PF, physical functioning; RF, role functioning; EF, emotional functioning; CF, cognitive functioning; SF, social functioning. * *p*-value from ANCOVA adjusted for age, marital status, caregiver, practicing a religion, job status, monthly household income, educational level, type of national health insurance, comorbidity, time since diagnosis, NSCLC stage, current and prior treatments, and histological type.

**Table 4:** Factors affecting functioning in patients undergoing treatment for non-small cell lung cancer (NSCLC).

Functioning Domains	Physical Functioning		Role Functioning		Emotional Functioning		Cognitive Functioning		Social Functioning	
Variables	Partial R^2^	β (SE)	Partial R^2^	β (SE)	Partial R^2^	β (SE)	Partial R^2^	β (SE)	Partial R^2^	β (SE)
Increasing lack of energy-related symptoms	0.22	−7.5 (2.0) ***	0.18	−11.6 (2.5) ****	―	―	―	―	0.05	−5.3 (2.2) *
Increasing pain-related symptoms	0.04	−4.9 (1.6) **	―	―	0.29	−12.2 (1.6) ****	0.05	−3.7 (1.2) **	―	―
Impaired concentration	―	―	0.03	−16.7 (7.4) *	―	―	0.28	−25.4 (4.4) ****	―	―
Higher age	―	―	―	―	―	―	―	―	―	―
Higher education	0.03	13.6 (4.2) **	―	―	―	―	―	―	―	―
Having a job	0.02	11.1 (5.2) *	―	―	0.03	14.5 (7.0) *	―	―	0.09	1.2 (0.3) **
Higher income	―	―	―	―	―	―	―	―	0.07	14.1 (6.7) *
Current radiotherapy	―	―	―	―	0.02	−16.3 (6.2) **	―	―	―	―
Current immunotherapy	0.06	−30.0 (12.1) *	―	―	―	―	―	―	―	―
Model R^2^	0.37	―	0.21	―	0.34	―	0.33	―	0.21	―

SE, standard error. **** < 0.0001, *** < 0.001, ** < 0.01, * < 0.05.

## Data Availability

Data available on request due to ethical restrictions.
